# Diatom evidence of 20th century ecosystem change in Lake Baikal, Siberia

**DOI:** 10.1371/journal.pone.0208765

**Published:** 2018-12-19

**Authors:** Sarah L. Roberts, George E. A. Swann, Suzanne McGowan, Virginia N. Panizzo, Elena G. Vologina, Michael Sturm, Anson W. Mackay

**Affiliations:** 1 Canada Centre for Inland Waters, Environment and Climate Change Canada, Burlington, Ontario, Canada; 2 School of Geography, University of Nottingham, Nottingham, United Kingdom; 3 Institute of Earth’s Crust, Siberian Branch of the Russian Academy of Sciences, Irkutsk, Russia; 4 Swiss Federal Institute of Aquatic Science and Technology EAWAG-ETH, Zürich, Switzerland; 5 Environmental Change Research Centre, Department of Geography, University College London, London, United Kingdom; Chinese Academy of Sciences, CHINA

## Abstract

Lake Baikal has been experiencing limnological changes from recent atmospheric warming since the 1950s, with rising lake water temperatures, reduced ice cover duration and reduced lake surface-water mixing due to stronger thermal stratification. This study uses lake sediment cores to reconstruct recent changes (c. past 20 years) in Lake Baikal’s pelagic diatom communities relative to previous 20^th^ century diatom assemblage records collected in 1993 and 1994 at the same locations in the lake. Recent changes documented within the core-top diatom records agree with predictions of diatom responses to warming at Lake Baikal. Sediments in the south basin of the lake exhibit clear temporal changes, with the most rapid occurring in the 1990’s with shifts towards higher abundances of the cosmopolitan *Synedra acus* and a decline in endemic species, mainly *Cyclotella minuta* and *Stephanodiscus meyerii* and to a lesser extent *Aulacoseira baicalensis* and *Aulacoseira skvortzowii*. The north basin, in contrast, shows no evidence of recent diatom response to lake warming despite marked declines in north basin ice cover in recent decades. This study also shows no diatom-inferred evidence of eutrophication from deep water sediments. However, due to the localised impacts seen in areas of Lake Baikal’s shoreline from nutrient pollution derived from inadequate sewage treatment, urgent action is vital to prevent anthropogenic pollution extending into the open waters.

## Introduction

In recent decades, lakes around the world have experienced increased surface water temperatures [1, 2]. The impacts of this warming are numerous but include changes in the vertical thermal structure of the water column and longer ice-free seasons, together with shifts in ecosystem structure and function [1, 3–6]. For example, lakes have experienced community compositional changes such as large-scale shifts in plankton, with increases in taxa (such as picocyanobacteria) which are better adapted to reduced turbulent mixing [7–12]. These changes are not restricted to small—medium sized water bodies. Very large lakes, including the Laurentian Great Lakes, Lake Tahoe (North America) and Lake Tanganyika (East Africa), have also experienced shifts in their biological communities and autochthonous primary production [2, 11–15]. At the same time, lake ecosystems are also sensitive to the impacts of climate warming on watershed processes, which can lead to alterations of fluvial inputs, including the flux of nutrients to lakes and the browning of lake waters from increased dissolved organic matter (DOM) input [9, 10, 16–18]. Such changes can influence autotrophic algal and bacterial communities through changes in nutrient cycling and light availability [19, 20].

**Fig 1:**
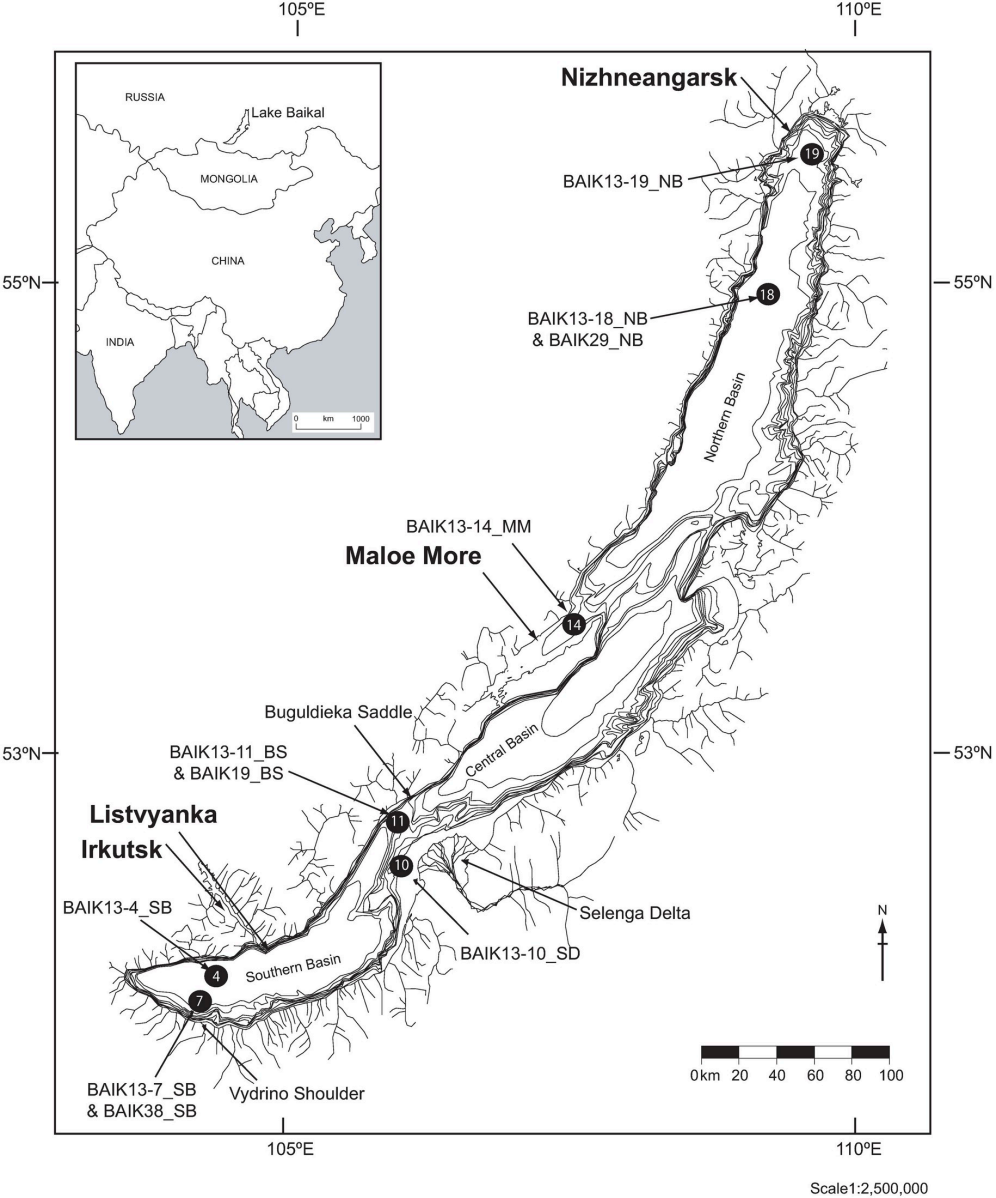
Map of Lake Baikal showing coring station sites across the south and north basin, and within Maloe More Strait, off the central basin. [SB: South basin, SD: Selenga Delta, BS: Buguldieka Saddle, MM: Maloe More and NB: North basin].

Lake Baikal, the world’s oldest, deepest, and most voluminous lake, is experiencing impacts from climate warming. Atmospheric temperatures in the region around Lake Baikal in southern Siberia have increased significantly in recent decades, with mean annual surface air temperatures in the nearby city of Irkutsk (Fig 1) rising from -5.8°C to 0.3°C over the last 20 years [21]. Atmospheric temperature data from a climate station in Nizhneangarsk (Fig 1), in the north basin catchment area of Baikal, similarly shows a less pronounced warming trend from -1.27°C to 0.48°C between 1952 and 2013 CE (mean annual temperatures; http://climexp.knmi.nl/). The scale of recent atmospheric warming, regionally, is unprecedented for the past 1000 years [22]. Concordant with recent warming, summer surface lake water temperatures have increased by over 2°C over the past 60 years [9, 23, 24], alongside seasonal changes to ice cover dynamics [25, 26]. Shorter ice cover and warmer water temperatures have led to marked increases in chlorophyll-*a* concentrations and summer plankton biomass in the south basin of Lake Baikal over the past 60 years [9, 23, 24, 27–29].

Further 21^st^ Century warming is predicted to trigger additional plankton community change with a shift from the production of endemic, heavily silicified diatoms towards lighter, littoral diatoms and autotrophic picoplankton (APP) [23, 30–33]. For example, [33] predicted that future reductions in ice duration associated with regional warming, would result in enhanced transport of diatoms such as *Stephanodiscus meyerii* and *Synedra acus* to pelagic regions, concomitant with the decline in endemic pelagic species such as *Aulacoseira baicalensis* and *Cyclotella minuta*. These changes may be enhanced by elevated spring run-off from increased precipitation [34], increasing silicon and other nutrient delivery to the lake, further encouraging the growth of species such as *Synedra acus* [33, 35], while also altering carbon cycling in the lake due to enhanced delivery of dissolved organic carbon (DOC) and particulate organic carbon (POC) [17, 23].

To date, these predictions at Lake Baikal have largely been made from diatom records collected in the early 1990’s [31, 33]. More recent diatom changes are examined here, as Lake Baikal has experienced limnological and biological changes with rising lake water temperatures and reductions in ice cover duration [9, 24, 25, 27–29]. Furthermore, it has become increasingly apparent that over the past decade cultural eutrophication from shoreline settlements has also impacted littoral regions of the lake [36, 37], giving rise to the potential for multiple stressors impacting its ecological resilience. In this paper, we test these predictions and assess whether the diatom flora in Lake Baikal has indeed undergone shifts in community composition in response to the major increases in global and regional atmospheric temperatures over the past 20 years [34]. In particular we examine whether there has been a transition from a flora dominated by heavily-silicified endemic species to one that is more cosmopolitan with lighter, less-silicified species [9, 23, 24, 27, 28, 33].

## Methodology

### Short cores

Short cores (< 65 cm) were collected in March and August 2013 from 7 coring stations across the southern and northern basins of Lake Baikal, as well as the Maloe More Strait (Fig 1). No permits were required as part of this work. All expeditions on Lake Baikal were organised by the Institute of Earth’s Crust, Siberian Branch of the Russian Academy of Sciences, Irkutsk, Russia. No endangered or protected species were put at risk during this project. The codes used to define the locations of these coring sites across Lake Baikal include south basin (SB), nearby the Selenga Delta (SD), at the Buguldieka Saddle (BS), Maloe More Strait (MM) and north basin (NB), and these have been applied after the original coring site codes. Cores were collected using a UWITEC corer with PVC-liners (Ø 63 mm), which provided complete and undisturbed recovery of the sediment/water interface. All coring stations were > 5 km from the shore with some sites coinciding with previous short cores taken in 1993 and 1994 –see Table 1 and [31] for details. Several cores were collected at each station. One core was sub-sampled in the field at a resolution of 0.2 cm and transported to the UK for diatom analyses and ^210^Pb radiometric dating, and at least one other core was transferred to the Institute of the Earth's Crust (Irkutsk) before being cut, photographed and lithologically described, based on smear slide inspection. A Bartington MS2E High Resolution Surface Scanning Sensor [38] was used for non-destructive measurement of magnetic susceptibility (MS), with a resolution of 1 cm and reproducibility of <5%.

**Table 1:** Location of both the sediment cores collected in March and August 2013 and the published diatom records at nearby coring sites in Lake Baikal collected in 1993/1994 [31]. Water depths at each coring site are shown, along with the location which they were collected from. [SB: South basin, BS: Buguldieka Saddle; SD: Selenga Delta, MM: Maloe More and NB: North basin].

Sediment cores collected in March and August 2013	Sediment cores published in [31]	Basin	N	E	Water depth (m)
BAIK13-4C_SB	(-)	South	51°41’33.8”	104°18’00.1”	1350
BAIK13-7A_SB	BAIK38_SB	South	51°34’06”	104°31’43”	1080
BAIK13-10A_SD	(-)	South/Selenga	52°11’07”	106°05’38”	66
BAIK13-11C_BS	BAIK19_BS	South/Selenga	52°27’00”	106°07’32”	345
BAIK13-14C_MM	(-)	Maloe More Strait	53°21’03”	107°29’88”	200
BAIK13-18A_NB	BAIK29_NB	North	54°47’31.4”	109°14’15.3”	890
BAIK13-19B_NB	(-)	North	55°38’57.8”	109°4657.7”	460

### Diatom analysis

Surface sediment samples (upper 2 cm) covering at least the past 30 years were analysed for diatoms, enabling overlap with published diatom records covering the interval prior to this [31]. These diatom records were combined based on independently derived ^210^Pb ages of the sediment samples from the cores collected in 2013 and in 1993/1994 [31]. Sample preparation followed previous protocols for diatom analysis on Lake Baikal sediment samples with no chemical treatments in order to minimise valve breakage, especially of lightly silicified diatom species [31, 39]. To calculate diatom concentrations (10^4^ valves/g dry weight), a known weight of divinylbenzene microspheres (approximately 1–2 g) was added to the cleaned samples [40]. Subsamples of the suspensions were diluted and settled out onto coverslips and fixed onto slides with Naphrax on a hotplate at 130°C. A total of 300 valves were counted at x1000 magnification by using an oil immersion lens and phase contrast under a Zeiss Axioskop 2 plus light microscope.

Diatom dissolution also plays a role in shaping sedimentary diatom assemblages, because some species are more heavily silicified (*A*. *baicalensis*) than others (*S*. *acus*) and consequently more resistant to dissolution [41]. Diatom dissolution was estimated by categorising endemic diatom species; *Aulacoseira baicalensis* (Meyer) Simonsen, *Aulacoseira skvortzowii* (Edlund, Stoermer and Taylor), *Cyclotella baicalensis* (Meyer) Skv., and *Cyclotella minuta* (Skv.) Antipova, into three stages of valve preservation. A diatom dissolution index (DDI) [42] was then calculated to quantify the extent of diatom preservation in every sample [43]. A DDI value of 0 indicates that all diatom valves are affected by dissolution, while a DDI value of 1 indicates that all the diatom valves are in a pristine condition. Indices have been combined to express the index as dissolution per sample. There is possibly a third endemic *Cyclotella* species, *Cyclotella ornata* [39] of intermediate size between *C*. *baicalensis* and *C*. *minuta*. However, [44] suggest that it most likely represents smaller valves of *C*. *baicalensis*, or occasionally *C*. *minuta* that has undergone size regeneration. Either way, in sedimentary material the centre areas of these valves are often indistinguishable from the central areas of *C*. *minuta* valves after breakage and dissolution. Here we group potential *C*. cf. *ornata* valves in with *C*. *minuta* (*C*. *minuta* agg.), which also makes comparisons with earlier studies, e.g. [31] consistent.

### Chronology

Dried sediments from cores BAIK13-4F_SB, BAIK13-7A_SB, BAIK13-10A_SD, BAIK13-11C_BS, BAIK13-14C_MM, BAIK13-18A_NB and BAIK13-19B_NB (Fig 1) were analysed for ^210^Pb, ^226^Ra, ^137^Cs and ^241^Am, by non-destructive direct gamma spectrometry [45] at the UCL Environmental Radiometric Facility, using an ORTEC HPGe GWL series well-type coaxial low background intrinsic germanium detector. Age-depth models were constructed using polynomial regressions fitted to the ^210^Pb data with additional degrees added until no improvement occurred in the fitted model under an ANOVA test at the 95% confidence interval.

### Statistical analysis

To constrain how diatom assemblages in the core-tops (representing material over the past c. 20 years) have differed through the 19^th^ and 20^th^ Centuries, squared chord distance (SCD) dissimilarity scores were determined using R [46] for cores BAIK13-7A_SB, BAIK13-11C_BS and BAIK13-18A_NB. These cores overlap with previously published cores BAIK38_SB, BAIK19_BS and BAIK29_NB respectively (Table 1), and the results can be used as a baseline to evaluate whether diatom assemblages have significantly altered since the 1990’s [31]. Ranging between 0 and 5, a SCD score of 0 indicates that the diatom assemblages in two samples are identical, whereas a score of 5 indicates samples are completely different in their diatom assemblage composition. To examine the timing of significant changes within the diatom datasets, breakpoint analysis was carried out on the SCD scores in *R* using the *segmented* library [46, 47]. Breakpoint analyses determines if there is a sharp change in the directionality of the dataset, by fitting two linear regression models, joined at a breakpoint. The significance of each breakpoint was also tested.

Temporal changes in the diatom assemblages were further visualised using principal components analysis (PCA) of major taxa in all core-top samples collected in 2013 together with data from the overlapping cores collected in 1993/1994 (Table 1). Following detrended correspondence analysis (DCA), which demonstrated a linear response based on the gradient length of the first axis, data were reanalysed using PCA with square root transformation of species abundances using Canoco 4.5 [48].

## Results

### Core lithology

**Fig 2:**
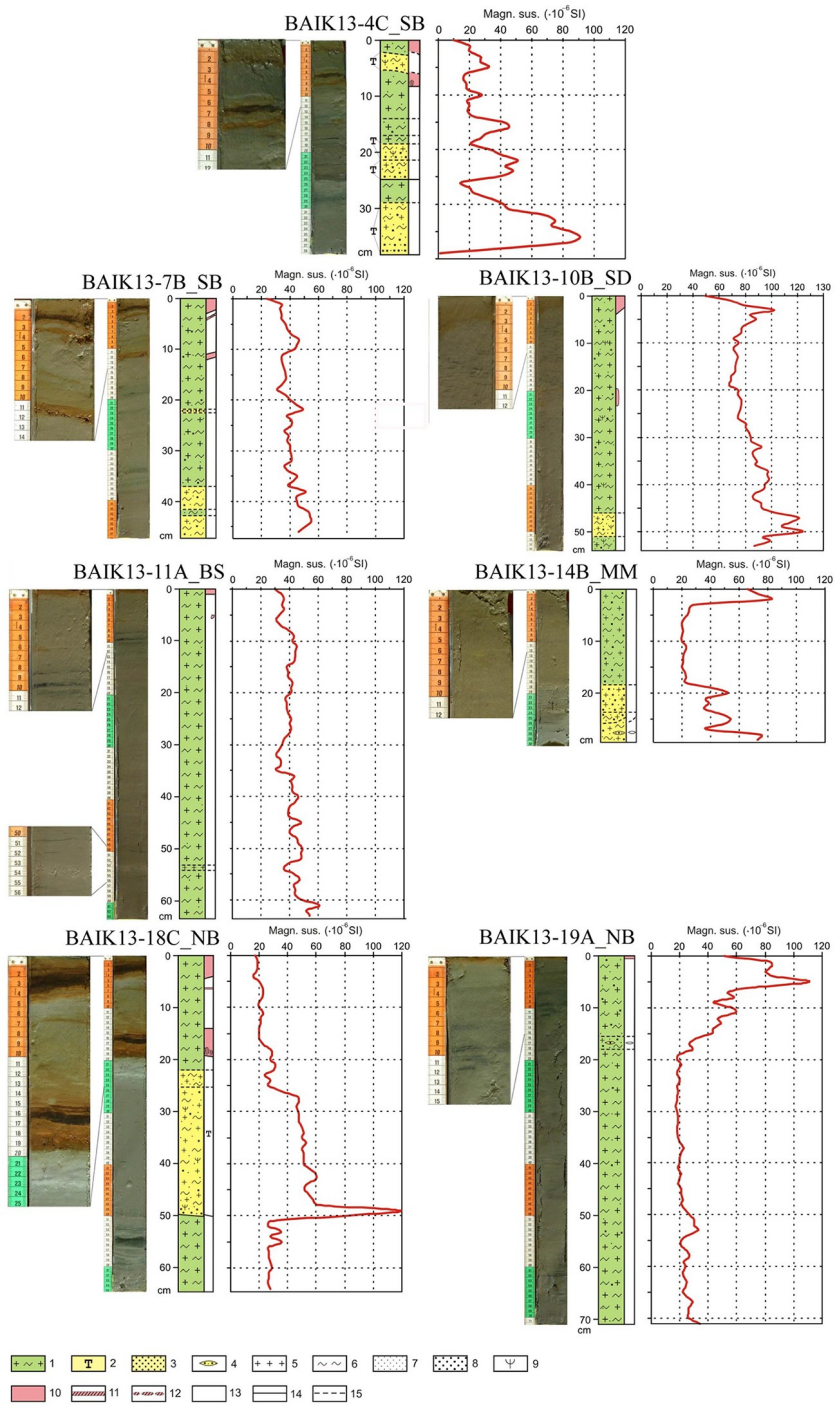
Core lithology and magnetic susceptibility profiles for BAIK13-4C_SB, BAIK13-7B_SB, BAIK13-10B_SD, BAIK13-11A_BS, BAIK13-14B_MM, BAIK13-18C_NB and BAIK13-19A_NB sediment cores. Lithology key: 1—pelagic mud, 2—turbidite, 3—sandy sediment, 4—diatoms, 5—clay, 6—silt, 7—sand, 8—land plant remains. Right column: 9—oxidized sediment, 10—Fe/Mn crust, 11—fragments of Fe/Mn crust, 12—O_2_ reduced sediment. Boundaries between layers: 13—distinct boundaries between layers, 14—indistinct boundaries between layers. The lithology for cores BAIK13-4C_SB and BAIK13-7B_SB have previously been published in [49]. [SB: South basin, SD: Selenga Delta, BS: Buguldieka Saddle, MM: Maloe More Strait and NB: North basin].

All cores were dominated by terrigenous material with varying levels of oxidation (the water column of Lake Baikal is fully oxygenated) (Fig 2). Turbidites were present in cores BAIK13-4C_SB and BAIK13-18C_NB, but the uppermost turbidites (2.0–5.3 cm in BAIK13-4C_SB; 22.0–49.8 cm in BAIK13-18C_NB) occurred below the samples analysed in this study and are therefore not discussed further.

### ^210^Pb age models

**Fig 3:**
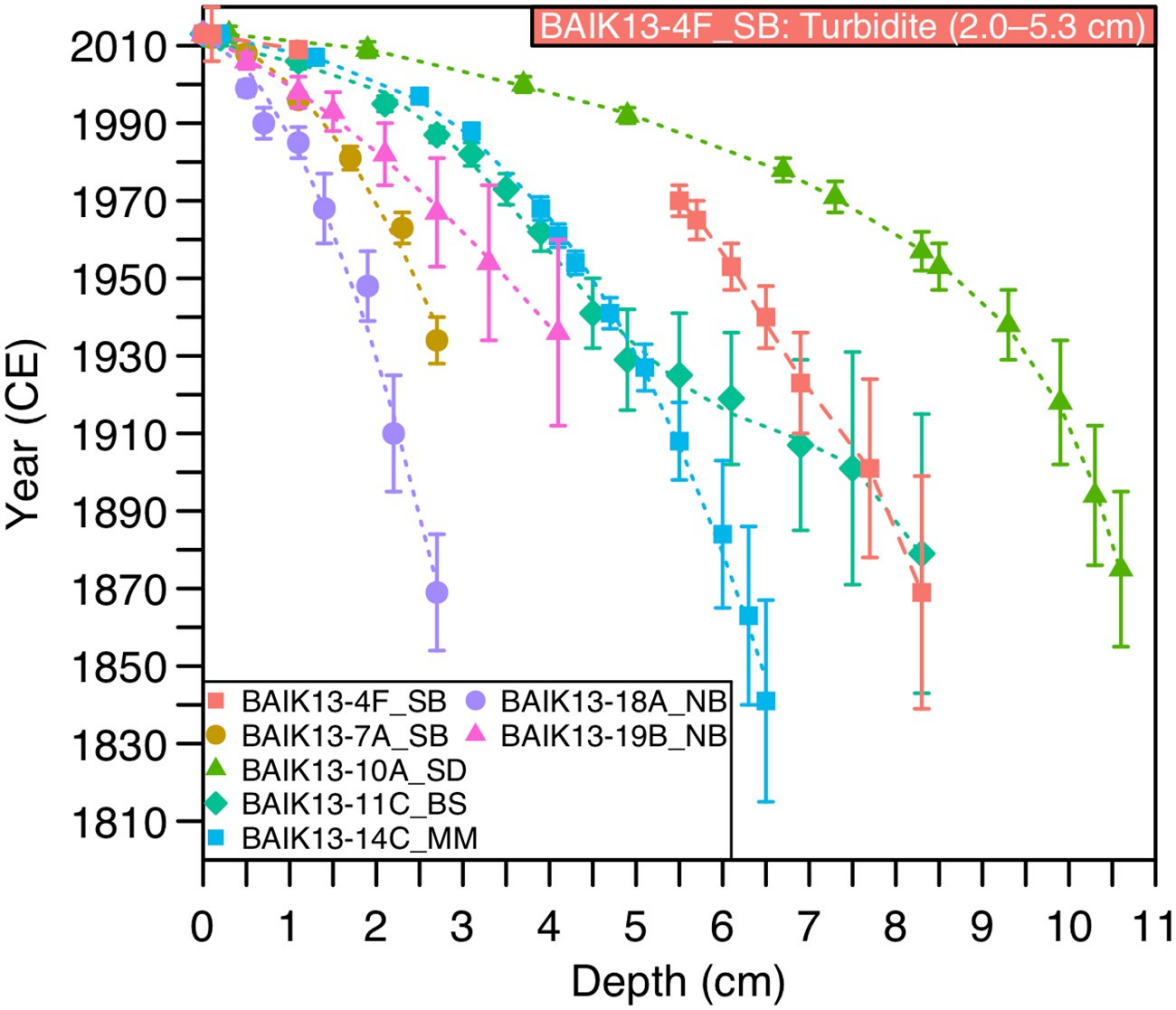
^210^Pb age models for BAIK13-4F_SB, BAIK13-7A_SB, BAIK13-10A_SD, BAIK13-11C_BS, BAIK13-14C_MM, BAIK13-18A_NB and BAIK13-19B_NB sediment cores. In the upper sediments, turbidites are present between 2.0–5.3 cm in the BAIK13-4F_SB sediment core. [SB: South basin, SD: Selenga Delta, BS: Buguldieka Saddle, MM: Maloe More and NB: North basin].

Total ^210^Pb activity reaches equilibrium with supported ^210^Pb at a depth of 9 cm (BAIK13-4F_SB), 4 cm (BAIK13-7A_SB), 13 cm (BAIK13-10A_SD), 10 cm (BAIK13-11C_BS), 7 cm (BAIK13-14C_MM), 3 cm (BAIK13-18A_NB) and 5.5 cm (BAIK13-19B_NB) (Fig 3). At all sites, ^210^Pb dates were calculated using the constant rate of ^210^Pb supply (CRS) model [50], and where possible, dates independently verified using ^137^Cs and ^241^Am data. For example, a well resolved ^137^Cs activity peak at 5.5–5.7 cm agrees with ^210^Pb dated sediments at BAIK13-4F_SB. At BAIK13-10A_SD, ^137^Cs activity shows a broad peak at 6.7–8.5 cm, and because ^241^Am peaks at 8.3 cm, this represents the 1963 CE (common era) peak in radioactive fallout; the ^210^Pb date at 1963 CE falls in this range. At BAIK13-11C_BS, ^210^Pb dating can be confirmed with peaks of ^137^Cs at both 1986 CE and 1963 CE. At BAIK13-14C_MM, high ^137^Cs activities in top 4.1cm concur with ^210^Pb dating that these sediments were deposited since 1963 CE. At BAIK13-18B_NB, the peak in ^137^Cs activity between 0.7–1.1 cm concurs with CRS ^210^Pb dating of 1986 CE being between 0.7–1.1cm, so this peak likely represents Chernobyl accident in 1986 CE. At only two sites, BAIK13-7A_SB and BAIK13-19B_NB, were ^137^Cs and ^241^Am activities not able to be used to confirm ^210^Pb dating. For all sites, the final age-depth model shows a good fit to the ^210^Pb dates with an adjusted R^2^ > 0.99 (Fig 3). Mean uncertainty in the individual ^210^Pb dates across all four cores ranges from 2–36 years is: BAIK13-4F_SB: $\bar{\text{x}}=8$, range = 2–30; BAIK13-7A_SB: $\bar{\text{x}}=3$, range = 2–6, BAIK13-10A_SD: $\bar{\text{x}}=7$, range = 2–20; BAIK13-11C_BS: $\bar{\text{x}}=12$, range = 2–36; BAIK13-14C_MM: $\bar{\text{x}}=8$, range = 2–26; BAIK13-18A_NB: $\bar{\text{x}}=8$, range = 2–15; BAIK13-19B_NB: $\bar{\text{x}}=11$, range = 2–24 (Fig 3).

### Diatom profiles

#### South basin sites

**Fig 4:**
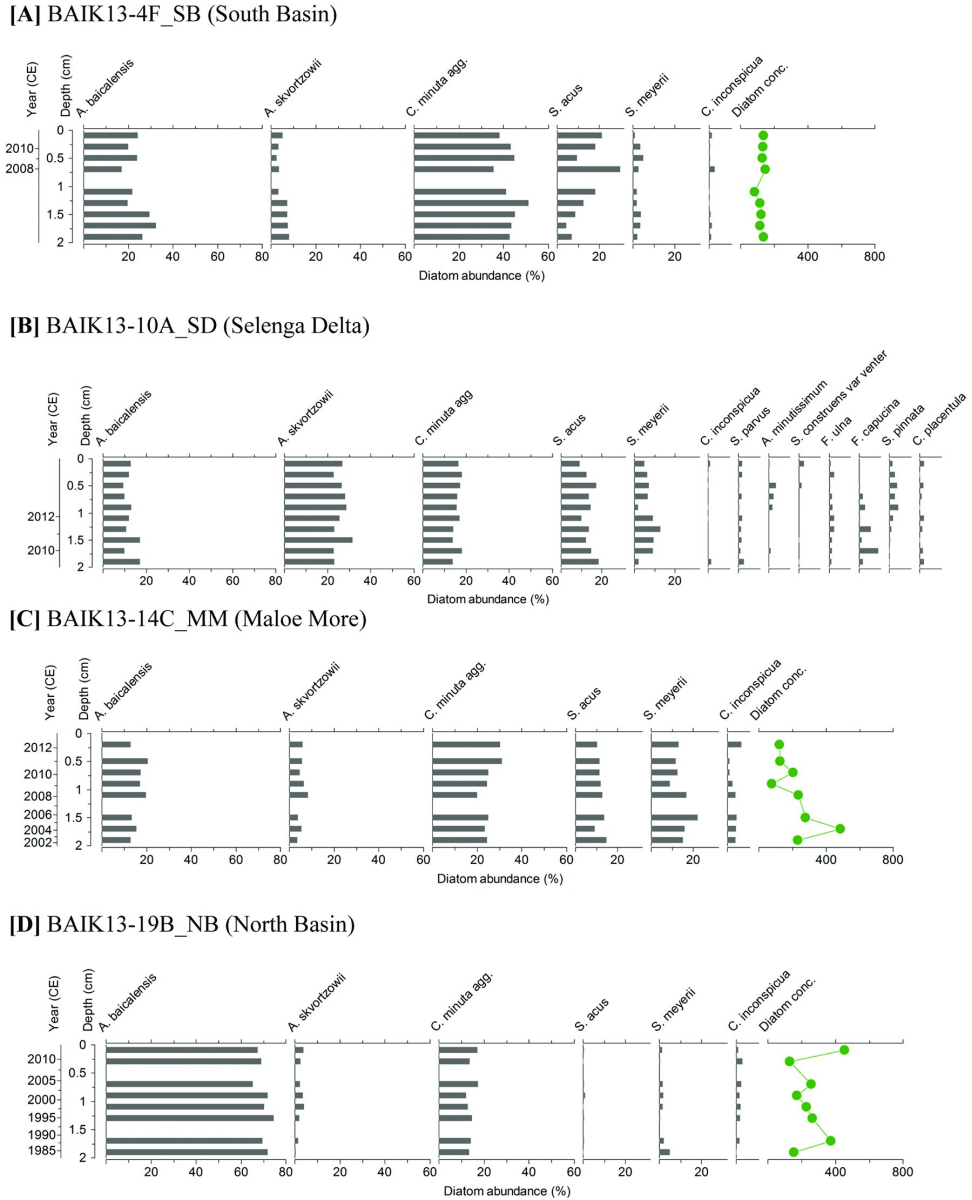
Stratigraphic plots of core top diatom assemblages are shown for (A) BAIK13-4F_SB, (B) BAIK13-10A_SD, (C) BAIK13-14C_MM, (D) BAIK13-19B_NB. Diatom concentrations (10^4^ valves/g DW) are shown alongside diatom abundances (species with > 2% abundance). [SB: South basin; SD: Selenga Delta; MM: Maloe More; NB: North basin].

In the south basin, at BAIK13-4F_SB (Fig 4) *S*. *acus* increased from 7% to 22% abundance over the top 2 cm of the core (2006–2013 CE). Over the same interval *A*. *baicalensis* declined from 26% to 24%, along with *A*. *skvortzowii* (decrease from 8% to 5%). *C*. *minuta* agg. varies between 35% to 51% relative abundance and *S*. *meyerii* varied between 2% to 5%. Diatom concentrations in the upper sediments ranged between 85.1 to 147.1 x 10^4^ valves/g DW over the last decade.

**Fig 5:**
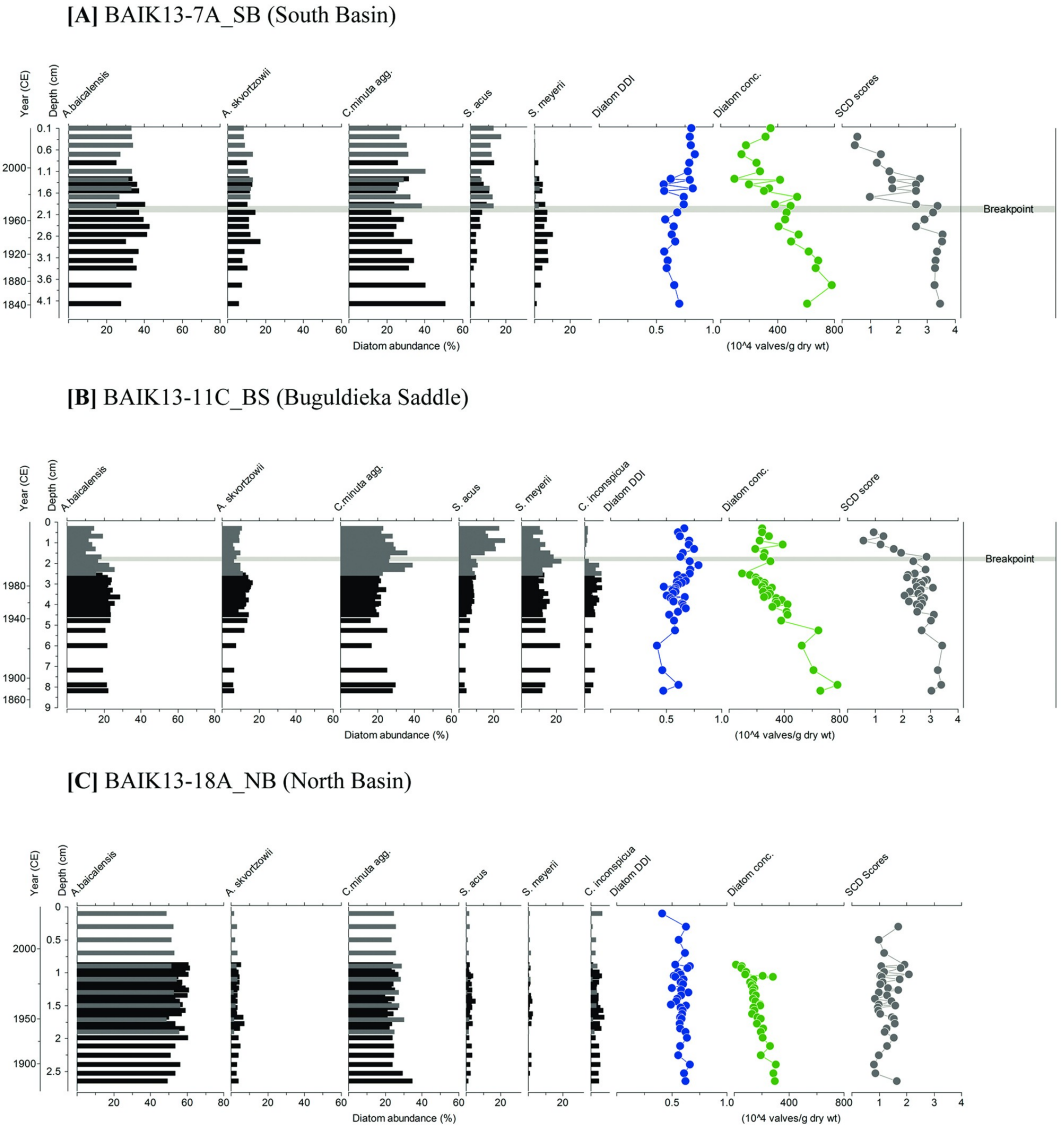
Stratigraphic plot of diatom assemblages at (A) BAIK13-7A_SB, (B) BAIK13-11C_BS, (C) BAIK13-18A_NB with core top samples (represented by grey bars) overlapping with diatoms records from [31] (A) BAIK38_SB, (B) BAIK19_BS, (C) BAIK29_NB (represented by black bars). Diatom Dissolution Index (DDI) and diatom concentrations (10^4^ valves/g DW) are shown alongside diatom abundances (species with > 2% abundance). SCD dissimilarity scores are shown for the diatom dataset. The timings of significant breakpoints in the SCD scores are highlighted in grey for BAIK13-7A_SB and BAIK13-11C_BS diatom assemblages. No significant breakpoints were found in the BAIK13-18A_NB diatom assemblage data. [SB: South basin; BS: Buguldieka Saddle; NB: North basin].

In the south basin, at site BAIK13-7A_SB (Fig 5) there was a good overlap with the core diatom assemblages from BAIK38_SB in [31] between 0.9–1.9 cm. *S*. *acus* relative abundances in the surface sediment assemblages from BAIK13-7A_SB ranged from 6% to 18%, *A*. *baicalensis* ranged from 25% to 41% relative abundance over the last c. 40 years between 1975–2013 CE, and *Cyclotella minuta* agg. ranged between 38% and 24%. Diatom concentrations ranged between 540.8 and 98.5 x 10^4^ valves/g DW over the last c. 30+ years (1980–2013 CE). Diatom dissolution index (DDI) values fluctuated between 0.5 and 0.8.

In the south basin, at site BAIK13-10A_SD (Fig 4), in the shallow waters off the coast of the Selenga Delta, the diatom assemblage diversity (> 2% abundance) was higher than the core tops in this study, with *Stephanodiscus parvus* (1–3% abundance), *Staurosira construens* var. *venter* (1–3% abundance), *Fragilaria ulna* (2–4% abundance), *Fragilaria capucina* (1–9% abundance), *Achnanthidium minutissimum* (1–3% abundance), *Staurosirella pinnata* (1–4% abundance) and *Cocconeis placentula* (1–2% abundance). Over the top 2 cm (2009–2013 CE), percentage abundances remained relatively consistent for *A*. *baicalensis* (range between 9–17%), *A*. *skvortzowii* (ranged between 22–31%), *C*. *minuta* agg. (ranged between 13–18%) and *S*. *acus* (ranged between 10–17%), while *S*. *meyerii* ranged from 13% to 2% abundance.

At site BAIK13-11C_BS, in the waters of the Buguldieka Saddle opposite the Selenga Delta (Fig 1), there was a good overlap between 2.4–2.6 cm with the diatom assemblages from BAIK19_BS in [31] (Fig 5). The upper 2.3 cm in BAIK13-11C_BS showed a decreasing trend in *A*. *baicalensis* to abundances of c. 14% and *C*. *minuta* agg. to abundances of c. 20% and increasing abundances of *S*. *acus* to abundances of > 20% over the last c. 20 years (1993–2013 CE). Diatom concentrations showed a decreasing trend towards the surface sediments, from concentrations of 648.5 x 10^4^ valves/g DW to 241.1 x 10^4^ valves/g DW over the last c. 80 years (1930–2013 CE). DDI values fluctuated between 0.4 and 0.7.

#### Maloe More Strait

In the Maloe More Strait at site BAIK13-14C_MM (Fig 4), *A*. *baicalensis* (range = 13–20%), *A*. *skvortzowii* (range = 3–8%), *C*. *minuta* agg. (range = 19–31%), S. *acus* (range = 9–15%) and *Crateriportula inconspicua* (range = 4–6%) remained relatively consistent in abundance over the 2 cm (2002–2013 CE). However, over the same interval *S*. *meyerii* ranged from 22–13% abundance, and diatom concentrations ranged between 78.7 and 486.2 x 10^4^ valves/g DW.

#### North basin sites

In the centre of the north basin, at site BAIK13-18A_NB (Fig 1) there was a good overlap between 0.8–1.9 cm with the assemblages from BAIK29_NB in [31] (Fig 5). Assemblages within the upper 2 cm of BAIK13-18A_NB showed a decreasing trend in *A*. *baicalensis* (from c. 60% to c. 50%) and *S*. *acus* ranged from c. 6% to 1% (Fig 5). *C*. *minuta* agg. ranged in abundance between c. 20% to 30% over the last c. 80 years (1930–2013 CE). DDI values fluctuated between 0.5 to 0.7 and before the surface sediments, diatom concentrations showed a decreasing trend, from concentrations of 129.5 to 12.4 x 10^4^ valves/g DW between c. 1876–1992 CE.

In the north of the north basin, at site BAIK13-19B_NB (Fig 4), *A*. *baicalensis* and *C*. *minuta* agg. varied between 65–74% and 12–17% respectively, while there was little change in both *A*. *skvortzowii* and *C*. *inconspicua* (1–4% and 2–3% respectively). *S*. *meyerii* showed a small decline from 5–1.3% over the top 2 cm (1984–2013 CE) with diatom concentrations ranging between 129.5 and 455.6 (10^4^ valves/g DW) over the last 30 years.

### Temporal changes in the diatom assemblages

DDI values are > 0.5 in all samples, suggesting that the majority of the valves that make it into the sedimentary record are relatively well preserved [41]. Squared chord distance (SCD) dissimilarity scores (Fig 5) showed that surface sediment diatom assemblages in the south basin (BAIK13-7A_SB) and Buguldieka Saddle (BAIK13-11C_BS) were significantly different from older diatom assemblages determined from BAIK38_SB and BAIK19_BS respectively. In the south basin, the significant change in SCD values occurred at c. 1970 CE (p < 0.001; Fig 5). At the Buguldieka Saddle, breakpoint analysis of the SCD scores showed that a significant shift to the modern-day assemblage occurred later, at c. 2000 CE (p value < 0.001) (Fig 5). In contrast, diatom assemblages have changed very little in the north basin over the past 60 years, with no significant breakpoints found in the SCD scores at BAIK13-18A_NB (Fig 5).

**Fig 6:**
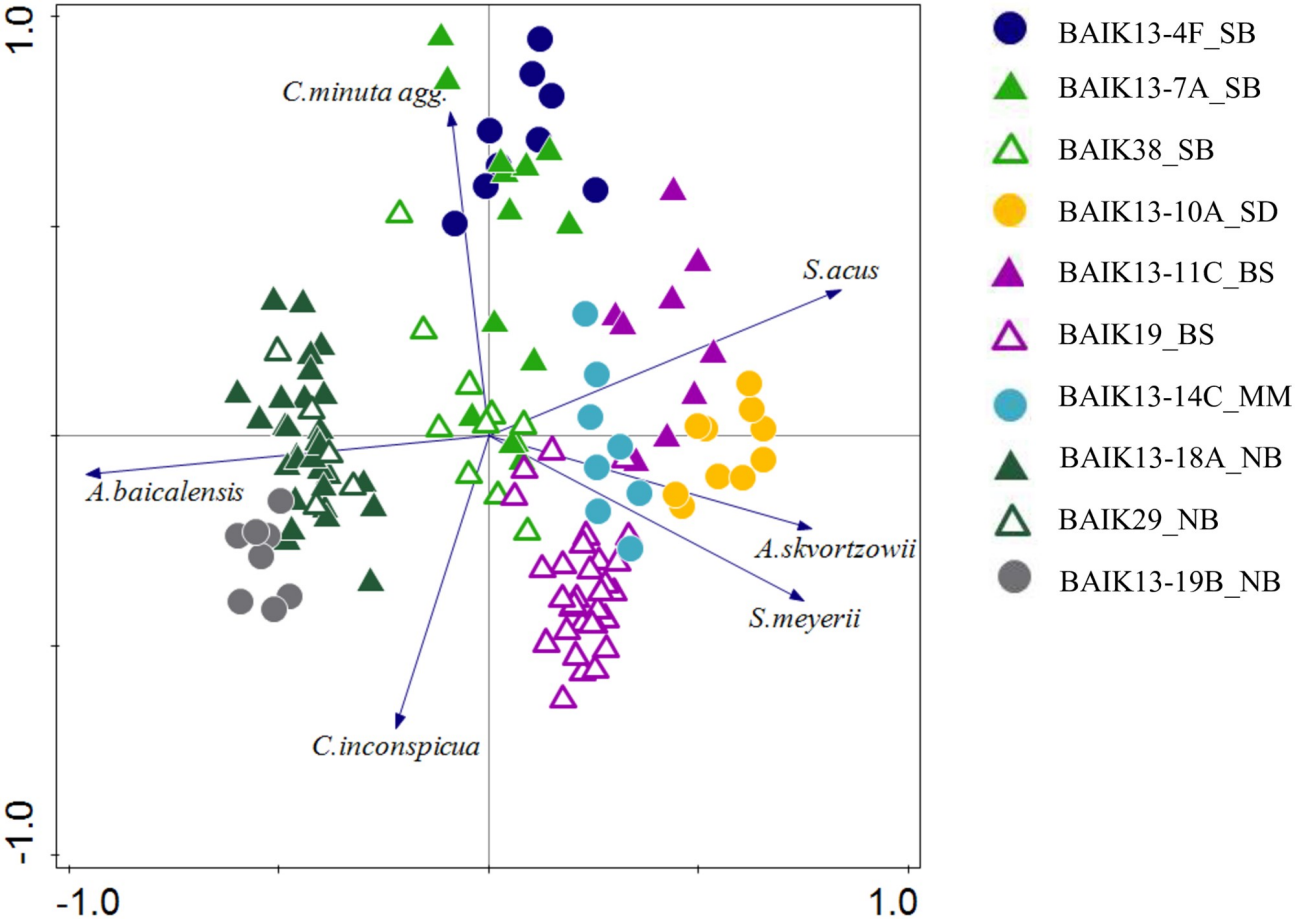
PCA of diatom species from all core samples. Core top samples (upper 2 cm) are from BAIK13-4F_SB, BAIK13-7A_SB, BAIK13-10A_SD, BAIK13-11C_BS, BAIK13-14C_MM, BAIK13-18A_NB and BAIK13-19B_NB sediment cores collected in 2013. The older sediments (20^th^ century sediments) are from cores BAIK19_BS, BAIK29_NB and BAIK38_SB [31]. Axis one eigenvalue is 0.47 and axis two eigenvalue is 0.24. [SB: South basin, SD: Selenga Delta, BS: Buguldieka Saddle, MM: Maloe More and NB: North basin].

PCA of all diatom assemblages investigated here revealed a difference between the core top samples analysed in this study and all samples in the older, overlapping cores from [31]. Core tops collected in 2013 contained higher abundances of *S*. *acus*, *A*. *skvortzowii* and *S*. *meyerii*, and lower abundances of *A*. *baicalensis* (Fig 6). Axis one explained 47% of the variance in the dataset and is driven largely by the pelagic species *A*. *baicalensis* (species score = +0.98) versus species linked to littoral habitats, mainly *S*. *acus*, *A*. *skvortzowii*, (and to a lesser extent *S*. *meyerii*). Axis two explained 24% of the variance in the dataset and is driven by a gradient of pelagic *C*. *minuta* agg. versus the smaller *C*. *inconspicua* species (Fig 6).

## Discussion

### Diatom ecology and spatial variability

The observed spatial distributions of the most common planktonic diatoms in Lake Baikal surface sediments (summarised by PCA in Fig 6) are linked to (i) complex interactions between lake morphology and chemistry, against a backdrop of a strong climatic gradient associated with the lake spanning over 4 degrees of latitude [31, 51], and (ii) evolutionary adaptations of different taxa over Quaternary timescales (e.g. [52]). PC1 reflects the gradient between *A*. *baicalensis* (which dominates north basin, deep-water surface sediments; species score = +0.98) and *A*. *skvortzowii* (-0.72), *S*. *acus* (-0.83) & *S*. *meyerii* (-0.79) (all associated with the relatively shallow-water surface sediments of the Buguldieka Saddle and the Maloe More Strait) (Fig 6). Unlike *A*. *baicalensis* abundances, Fig 5 reveals that the latter three species have never been abundant in the north basin for at least the last 100 years.

Both *A*. *baicalensis* and *A*. *skvortzowii* thrive in cold water temperatures below 5°C; they bloom mainly during the spring after ice break-up, and decline in abundance when lake waters warm to more than 5–6 °C [44, 53]. To avoid lethal, higher surface-water temperatures each species has evolved very different strategies [44, 54]. *A*. *baicalensis* grows well in conditions of low light [54], and due to its physiological plasticity competes well under ice through the production of smaller-sized valves, which then sink slowly after ice break up. After ice break up, mixing depths increase and declining light levels induce *A*. *baicalensis* to form longer, thicker resting cells full of storage products that allow them to survive throughout the period of summer stratification in cooler waters of intermediate depth (c. 50–100 m) [44]. Formation of the resting cells requires a lot of silica, which is met through remineralisation of silica from dissolving diatoms at moderate depths in the water column [44, 55]. *A skvortzowii* also avoids higher surface-water temperatures through the production of resting stages, but unlike *A*. *baicalensis*, its cue for the production of resting spores is phosphate utilisation by other algae (e.g. picoplankton) [53]. These non-siliceous algae effectively compete for nutrients [56], making them unavailable for most diatoms growing in the open lake. In the north basin of Lake Baikal however, lower Spring primary production (e.g. [24, 57]) means that phosphate concentrations rarely fall below the threshold level of 15–23 μg/L needed to induce *A*. *skvortzowii* spore formation [53]. Moreover, when phosphate concentrations do decline below the threshold level, it is often after isothermal mixing, meaning that growth of *A*. *skvortzowii* in the north basin is poor [53]. Elsewhere in Lake Baikal, *A*. *skvortzowii* does well because it has evolved planktonic and littoral life history stages, such that viable spores when produced remain in coastal sediments down to a depth of 25 m, where they can be resuspended by strong autumn wind-driven waves into the pelagic zone (ibid.) in time to bloom the following spring.

*S*. *acus* is a finely silicified, needle-shaped, cosmopolitan diatom, with high growth rates and low cell volumes [58]. It currently forms an important component of the under-ice diatom flora in Lake Baikal, and while reasonably abundant in both the south and central basins [31, 33] and shallow water regions (this study) it is almost absent from the north basin (Fig 6). *S*. *acus* is associated with high dissolved silica concentrations [34, 59], which may explain its negative relationship with *A*. *baicalensis* (Fig 6); recent work has shown that increased silicic acid availability is strongly controlled via population changes in diatom taxa [21, 58]. In particular, when increased populations of *A*. *baicalensis* result in “*Melosira* bloom years”, the availability of silicic acid for other species to uptake (such as *S*. *acus*) declines [58].

*S*. *meyerii* is a small endemic diatom which has a high temperature optimum of 15–17.5 °C [58], and like other *Stephanodiscus* species likely has a high affinity for phosphorus [34], although its autecology is poorly known. These adaptations likely account for the observed distributions (Fig 6), especially in regions which have high phosphorus loadings [33], such as the shallow waters of the Maloe More Strait, and off the coast of the Selenga Delta [60].

*C*. *minuta* is an endemic, co-dominant of the pelagic diatom community in both the north and south basins, although it mainly occupies a different temporal niche than the spring blooming diatoms, which accounts for it being positioned orthogonal to taxa associated with PCA axis 1 (Fig 6). Populations of *C*. *minuta* also grow under ice during spring, but their main growth occurs during autumn overturn [58]. Indeed, they are the only pelagic diatom to bloom in substantial numbers during the autumn in Lake Baikal; they persist in the upper water column for longer because they can tolerate water temperatures up to 11°C, so that when stratification breaks down at the end of the summer, and nutrient overturn occurs in the photic zone, cells are retrained first, giving them a competitive advantage [58]. However, at the finer scale, *C*. *minuta* abundances are not truly independent of spring blooming species. For example, when *A*. *baicalensis* blooms are particularly large, dissolved silica becomes depleted for all other diatoms, causing the subsequent autumnal crop of *C*. *minuta* to be much smaller [58]. Unfortunately, we know very little about the ecology of the endemic *C*. *inconspicua*, and currently can only describe its spatial distribution here as being present in very low relative abundances (< 2%) in the south basin and in the shallow waters off the Selenga Delta, but is persistent (> 2%) in the north basin and the Buguldieka Saddle, which accounts for its strong negative association with axis 2 in Fig 6.

### Environmental trends and temporal variability

**Fig 7:**
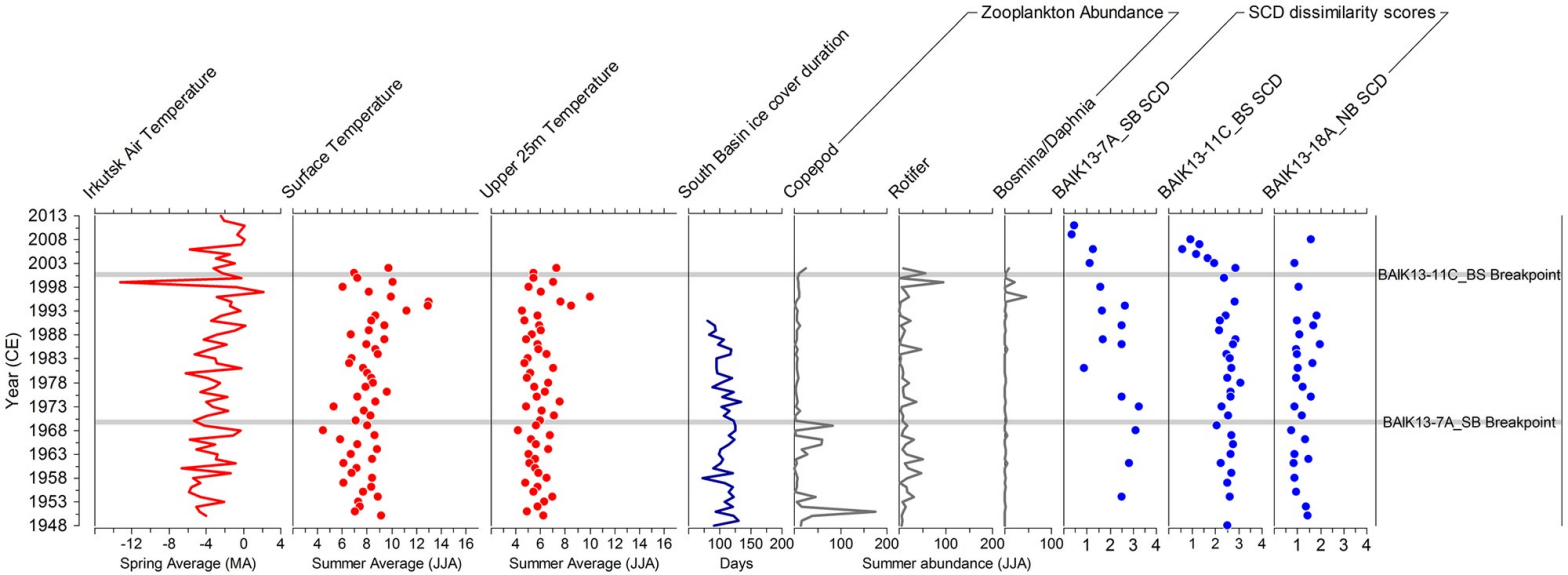
Regional spring Irkutsk air temperatures (March and April average) from a climate station next to the south basin of Lake Baikal (World Meteorological Organisation station 30710; 52°16’20”N, 104°18’29”E; elevation = 467 m) are shown over the period 1950 CE to 2013 CE. Summer surface water temperatures and water temperatures in the upper 25m of the water column (July to August average) are shown for the south basin over the period 1948 CE to 2002 CE [9]. Ice cover duration data for the south basin of Lake Baikal is shown between the period 1869 CE to 1996 CE [25]. Zooplankton summer abundance (1000 cells L^-1^) for copepods, rotifers and *Bosmina*/*Daphnia* are shown for the period 1950 CE to 2002 CE (data provided by S. Hampton). The stratigraphic plot includes SCD dissimilarity scores, comparing upper core top samples to all samples below in the same core. Timings of significant breakpoints in the SCD scores are also highlighted in grey for BAIK13-7A_SB and BAIK13-11C_BS diatom assemblages. No significant breakpoints were found in the BAIK13-18A_NB diatom assemblage data. [SB: South basin, BS: Buguldieka Saddle and NB: North basin].

Spring (March to April) air temperatures, from the KNMI Climate Explorer database (http://climexp.knmi.nl/) for Irkutsk, close to the south basin of Lake Baikal (World Meteorological Organisation station 30710; 52°16’20”N, 104°18’29”E; elevation = 467 m) increased between 1950–2013 CE (Fig 7). Air temperatures in the south basin of Lake Baikal have increased by c. 1.2°C per year, significantly higher than global trends, with greatest warming occurring during winter months, especially at the start of the 1950s and again since the early 1970s [21]. Increasing air temperatures have resulted in increases in average annual surface water temperatures of c. 2°C across the entire lake between 1977–2003 CE [24], with warming in the south basin, reaching increases of over 2.4°C during late summer months [9] and warming also reported in the north basin from 1977 to 2003 CE [24]. Warmer atmospheric temperatures have also resulted in marked changes in ice dynamics across the lake; annual ice duration in the south and north basins has declined, as has ice thickness since the start of the 1970s in the south basin [21, 25]. Since the 1950s, increased surface water temperatures have resulted in extended summer stratification [27], and increased chlorophyll-*a* concentrations [23, 24]. Total annual river inflow into the lake has increased over the past 100 years due to increased precipitation, bringing with it supplies of nutrients and dissolved silica [21]. A key question therefore, is whether this significant and unprecedented regional warming in southern Siberia [21, 22], led to a measurable impact, either directly or indirectly, on diatom community composition in Lake Baikal in particular.

Breakpoint analyses reveal that a significant change in diatom assemblage composition in the south basin was already underway by the early 1970s (Fig 5), in line with diatom changes seen in other temperate lakes [61, 62]. Although this trend was initially identified in the diatom record by [31], the more recent records show the disappearance of *S*. *meyerii* altogether from (BAIK13-7A_SB) in the south basin. At the Buguldieka Saddle (BAIK13-11C_BS), significant changes occurred c. 2000 CE, a few decades later than the more southern site. Here, the increase in *S*. *acus* is accompanied by wider changes in the diatom flora (Fig 5); again *S*. *meyerii* declines to low relative abundance (although it persists in record), while *C*. *inconspicua* almost disappears from the record. Both sites also show declines in heavily silicified species such as *A*. *baicalensis* and *C*. *minuta*. These changes are in contrast to no significant changes in the diatom flora from the north basin over the past 100 years (BAIK13-18A_NB).

Significant change in diatom SCD scores in the south basin core BAIK13-7A_SB (Fig 5) at c.1970 CE occurs soon after significant change in rising summer (July to August) surface water temperatures in the south basin (Fig 7; breakpoint at 1966 CE in the temperature record; p value < 0.001) and the start of declining ice thickness in the south basin [25, 26]. Phytoplankton monitoring studies from the south basin show major changes in the diatom flora consistent with those observed in the stratigraphic record. For example, within phytoplankton samples from the south basin, *A*. *baicalensis* concentrations have declined from c. 5 to 3 cells L^-1^ between 1950–2010 CE [29]. These changes are likely related to ice cover dynamics and increasing surface water stratification [27]. For example, the mixing depths in the south basin are deepening with lake warming, due to the higher summer surface water temperatures causing a stronger thermal stratification during the summer months [9, 27], which likely alters the position of resting cells within the water column, and their subsequent ability to be retrained into the photic zone. For *C*. *minuta*, this would mean fewer cells being entrained back up into the photic zone during the autumnal overturn, hence the declining valve numbers observed in the south basin (Fig 5).

Factors causing the heavily silicified diatoms to decline in abundance may also contribute to observed increases in *S*. *acus*. *S*. *acus* has a high temperature optima and fast growth rates [63], so can take advantage of rapidly warming surface waters after ice break-up. It is also able to grow through the summer months (e.g. [41, 57]) due to its low biovolume and higher surface—volume ratio, enabling it to stay in the photic zone longer than heavily silicified endemic species. So as heavily silicified, endemic diatom species find growth in increasingly warmer waters of southern Lake Baikal challenging, the cosmopolitan *S*. *acus* is able to flourish under these conditions, especially if availability of dissolved silica also increases [58].

An additional, notable, finding of this study is the disappearance in the endemic *S*. *meyerii* off the Vydrino Shoulder (BAIK13-7A_SB) and its marked decline at the Buguldieka Saddle (BAIK13-11C_BS) post c. 2003 CE (Figs 1 and 5). Its decline may be linked to changes towards reduced nutrient availability (including silicon and phosphorus concentrations). As mentioned above, *S*. *meyerii* may be indicative of more nutrient rich waters. However, in core BAIK13-10A_SD (Fig 4) within the shallow waters off the Selenga Delta, *Stephanodiscus parvus*, a cosmopolitan diatom which is often indicative of cultural enrichment, remains only at low abundance while *S*. *meyerii* declines in abundance over the past 10 years. High abundances of *S*. *acus* are seen within the BAIK13-10A_SD core top sediments, which are similar to those observed in BAIK13-4F_SB, BAIK13-7A_SB, BAIK13-11C_BS and BAIK13-14C_MM core tops (Figs 4 and 5). We suggest therefore that *S*. *acus* may be out-competing *S*. *meyerii* at these locations.

The lack of diatom changes in north basin sediments (BAIK13-18A_NB) contrast with those not only from the south basin and Buguldieka Saddle (BAIK13-7A_SB and BAIK13-11C_BS) (Fig 7), but also from many other lakes in cold regions (e.g, [61, 64]). Diatom responses to anthropogenic climate change are complex, involving interactions of multiple drivers, including changes to ice duration and variability, changes to light penetration from snow and ice cover, changes to timing and strength of surface water stratification, and changes to nutrient inputs, predation and disease, amongst others. One of the most important controls on diatom growth in Lake Baikal is light; for example, *A baicalensis* has evolved to take advantage of relatively low light intensities (under 40 μmol m^-2^ s^-1^) which can occur when cells are either vertically mixed by convection to depths in excess of 100m depth [54], or when snow cover persists on the frozen lake before ice break-up [65]. Given that seasonal snow cover on the north basin of Lake Baikal is much thicker than the rest of the lake (e.g. [33, 66]), light becomes a limiting factor, making the north basin much less favourable for diatoms to flourish during spring turnover (e.g. [54]). *A*. *baicalensis* is able to grow due to its evolutionary adaptations to low light intensities [54, 65], while *C*. *minuta* is able to grow because it blooms mainly during the autumn overturn. We conclude that the magnitude of change in snow and ice on the frozen north basin has not yet been sufficient to induce major changes in diatom communities in the north basin.

In addition to direct climate change, changes in the Lake Baikal diatom community may also be driven by changes within the lake’s food-web. Since the 1950s, increased surface water temperatures have resulted in changes to zooplankton numbers, including declining abundances of planktonic rotifers, but increased abundances of cladocera [9] and copepods [24]. With shorter ice cover duration on the lake, a rise in summer zooplankton biomass (copepods and *Bosmina/ Daphnia*) has been seen over the last 60 years [9] (Fig 7), with many of these groups also shifting to more shallow waters in the water column for reasons not yet fully understood [27]. Increasing zooplankton biomass (largely the endemic copepod *Epischura baicalensis*) in the upper 50 m of the water column in the south basin have also been determined [29] from 1950–2010 CE. The diatom assembly shifts seen in the south basin and Buguldieka sediment core (BAIK13-11C_BS) (Fig 5) could therefore also be affected by increased grazing pressures, both from increasing zooplankton numbers and their shift to shallow water positions, but also an increase in the spatial overlap between phytoplankton and copepods [27, 67]. For example, *C*. *minuta* is a key food source for the zooplankton *Epischura baicalensis* (e.g. [27, 33, 58]), while the larger endemic *Cyclotella baicalensis* although too large to be consumed by *E*. *baicalensis* is instead an important food source for the larger endemic gammarid *Macrohectopus brankcii* [58].

Finally, we find no evidence in our recent palaeolimnological records to suggest that local anthropogenic pollution has had a detectable effect on planktonic diatom communities. Nevertheless, in the past decade, evidence of cultural eutrophication of benthic and littoral regions of Lake Baikal near towns and tourist resorts is clear [36, 37]. Very poor or non-existent sewage treatment has resulted in localised high levels of nutrients (phosphorus & nitrogen) and organic matter [68]. Such eutrophication is causing large blooms of filamentous green algae to form, often far from local sources of pollution (including *Spirogyra* spp. *and Stigeoclonium tenue*), which then rot along the coastlines [36, 37, 69]. As yet there is no chemical monitoring evidence to suggest that littoral eutrophication has spread into the pelagic regions of lake (e.g. [68]), nor of a biological impact in the form of increased chlorophyll concentrations or decline in water transparency [24]. However, near-shore nutrient pollution can act as a precursor of off-shore disturbance, especially food-web dynamics [70]. Improvements to water treatment facilities in Lake Baikal are essential as soon as possible to ensure that eutrophication does not reinforce the negative impacts of anthropogenic climate change [71] on the Lake Baikal ecosystem.

## Conclusions

Palaeolimnological records of changing diatom assemblages over recent decades agree well with phytoplankton monitoring studies; numbers of heavily silicified diatom species in the south basin of Lake Baikal are in decline at the expense of increasing cosmopolitan, lighter, littoral species. These changes are consistent with previous predictions as to what might happen to diatoms in Lake Baikal as global mean temperature continue to increase [23, 33]. Warmer surface waters and increased period of stratification are very inhospitable for endemic taxa such as *A*. *baicalensis*, A. *skvortzowii* and *C*. *minuta*, while changes in nutrient availability may be restricting the growth of smaller, lighter endemics such as *S*. *meyerii*. Interactions with increasing numbers of primary consumers, especially endemic copepods such as *Epischura baicalensis* and amphipods such as *Macrohectopus*, are as yet undefined, but likely to be important. *S*. *acus* on the other hand may be benefiting from a combination of different impacts including shorter ice duration, longer periods of summer stratification, and increased dissolved silica availability from both increased river discharge, but also declining numbers of heavily silicified diatoms. At the moment these changes are confined to the south basin of Lake Baikal, and we have no evidence of warming impacts yet on sedimentary diatom assemblages in the north basin, most likely due to the persistence of unfavourable growing conditions. We also have no evidence in our records of increased impact from littoral eutrophication—however, given that littoral regions can act as early indicators of future wide-spread change, urgent action is still needed to stop nutrient pollution from entering the lake. Observed changes in the diatom flora are likely to be affected by several interacting factors which are still in play today, and much more work still needs to be done to unravel these multiple stressors.
