# Correction: Diatom evidence of 20th century ecosystem change in Lake Baikal, Siberia

**DOI:** 10.1371/journal.pone.0213413

**Published:** 2019-02-28

**Authors:** Sarah L. Roberts, George E. A. Swann, Suzanne McGowan, Virginia N. Panizzo, Elena G. Vologina, Michael Sturm, Anson W. Mackay

S1 File is omitted from the list of Supporting Information. It can be viewed below.

## Supporting information

S1 File Diatom data. (XLSX)
